# Short Course Radiation in the Treatment of Localized Rectal cancer: A Systematic Review and Meta-Analysis

**DOI:** 10.1038/srep10953

**Published:** 2015-06-09

**Authors:** Cui Chen, Peng Sun, Jian Rong, Hui-Wen Weng, Qiang-sheng Dai, Sheng Ye

**Affiliations:** 1 Department of Oncology, The First Affiliated Hospital of Sun Yat-sen University, Guangzhou, China; 2 Department of Medical Oncology, Sun Yat-sen University Cancer Center; State Key Laboratory of Oncology in South China; Collaborative Innovation Center for Cancer Medicine, Guangzhou, China; 3 Department of Extracorporeal circulation, The First Affiliated Hospital of Sun Yat-sen University.

## Abstract

This meta-analysis sets out to systematically assess the efficacy of short course radiation (SRT) for rectal cancer patients based on randomized, controlled trials. Eight randomized controlled trials involving 6894 patients were ultimately included in this meta-analysis. Three trials (n = 2574) compared SRT with surgery alone. Local recurrence was improved (HR = 0.48, 95% CI 0.40 to 0.58). Overall survival was marginally improved with an HR of 0.90 (95% CI 0.81 to 1.00), but the magnitude of benefit was heterogeneous across trials. An additional three trials (n = 3682) compared SRT with selective postoperative radiation ± chemotherapy. A significant reduction of local recurrence (HR = 0.44, 95% CI 0.35 to 0.56) was also found after SRT. However, no benefit in overall survival was observed. Moreover, two trials (n = 638) compared SRT with long course chemoradiation. There was no statistically significant local recurrence or overall survival difference observed between the two strategies. Patients receiving SRT had lower grade 3 or 4 acute treatment related toxicity (RR 0.11, 95% CI 0.05 to 0.22) whereas no difference in late toxicity was observed. Overall, SRT is a reasonable alternative for resectable rectal cancer patients and should be part of an informed discussion of treatment options for this group of patients.

Colorectal cancer has been ranked as the third most common cancer in the world^1^. Radical surgery is the mainstay of therapy for resectable colorectal cancer. Unlike colon cancer, however, the risk of local recurrence is higher in patients with rectal cancer^2^. The addition of radiation to surgery has been successfully used to improve local control^3^,^4^. There are two radiation regimens typically used in clinical practice: short course radiation (5 fractions of 5 Gy delivered over 1 week) and long course radiation (25–30 fractions of 1.8 or 2 Gy over 5–6 weeks).

Most randomized trials of short course radiation have been performed in the United Kingdom, Scandinavia and the Netherlands^5^,^6^,^7^,^8^. As the smaller number of fractions makes short course radiation (SRT) less expensive and more convenient than long course radiation, this schedule is preferred in these countries. However, it is not favoured in certain other European countries and North America. Unlike long course radiation combined with chemotherapy (CRT), SRT cannot be safely combined concurrently with adequate doses of systemic chemotherapy. The value of introducing a short course of preoperative radiotherapy before surgery for rectal cancer is still subject for debate. Among the unanswered questions are the effect of short course radiation on local recurrence or overall survival and, in particular, the relative merits compared with long course radiation combined with chemotherapy or other adjuvant approaches.

Meta-analysis provides a useful tool by which to summarize and analyse a body of research, particularly when apparent benefits are marginal or research results are conflicting^9^. No comprehensive meta-analysis on randomized trials related to short course radiation in rectal cancer has been performed to this point. Therefore, the current meta-analysis will provide an up-to-date exhaustive summary on the role of short course radiation in localized rectal cancer, including comparisons with surgery alone and other adjuvant approaches.

## Methods

### Aims

The specific aims were to systematically review the published literature of randomized controlled trials, comparing the following therapies: short course radiation versus surgery alone; short course radiation versus selective postoperative radiation ± chemotherapy; and short course radiation versus long course radiation combined with chemotherapy.

### Search Strategy

Trials were identified by searching PubMed, EMBASE, Chinese Biomedical Literature Database and the Cochrane library. Moreover, the important trial registries and conference proceedings (ASCO, ASCO GI, ECCO, ESTRO and AACR) were also searched. The following medical subject heading (MeSH) terms and key words were variably combined: rectal cancer, rectal carcinoma, neoadjuvant, chemoradiotherapy, radiation, short course radiation, randomized and trial. We used the “related articles” function to broaden the search. The deadline for trial inclusion was publication by June 30, 2014. The reference lists from the selected articles were also scanned for additional relevant trials. Each study was subjected to a quality assessment using a 14-point checklist by two investigators^10^.

### Selection Criteria

Two independent reviewers assessed the eligibility of abstracts identified by the search. If the eligibility was unclear from the abstract, the full article was retrieved for clarification. Any disagreements were resolved by discussion. The inclusion criteria were randomized controlled trials including patients with localized rectal cancer treated with short course radiation. Studies that were nonrandomized, were single-arm phase II trials, or had inadequate statistical analysis or information missing were excluded.

### Statistical Analysis

The data extracted from the trials were entered into RevMan version 5.2 software (Cochrane Collaboration, Oxford, United Kingdom) for meta-analysis to calculate the pooled estimates. Meta-analyses were conducted on local recurrence (LR), overall survival (OS) and toxicity. The results regarding LR and OS were analysed by calculating the pooled hazard ratio (HR) estimated from published trial data or from curve analysis, as they were both censoring and time to an event data^11^ In particular, we determined the value of observed minus estimated number of event (O–E) and its variance for each trial. The results are expressed as HRs with 95% CIs. An HR > 1.0 indicates that patients receiving SRT had a higher probability of experiencing an event. The results for treatment related toxicity were expressed as risk ratio (RR) with 95% CIs using the Mantel–Haenszel method^12^.

Heterogeneity was evaluated using Q statistics and quantified by the I^2^ statistic^13^,^14^. If the P value was less than 0.10 or I^2^ was greater than 50%, there was statistically significant between-study heterogeneity. Forest plots were used to display hazard ratios and risk ratios within individual trials. Visual examination of the funnel plots was performed to evaluate the likelihood of publication bias^15^. A two-sided P-value of <0.05 was considered significant for all analyses except the heterogeneity tests.

## Results

### Eligible studies

**Figure 1:**
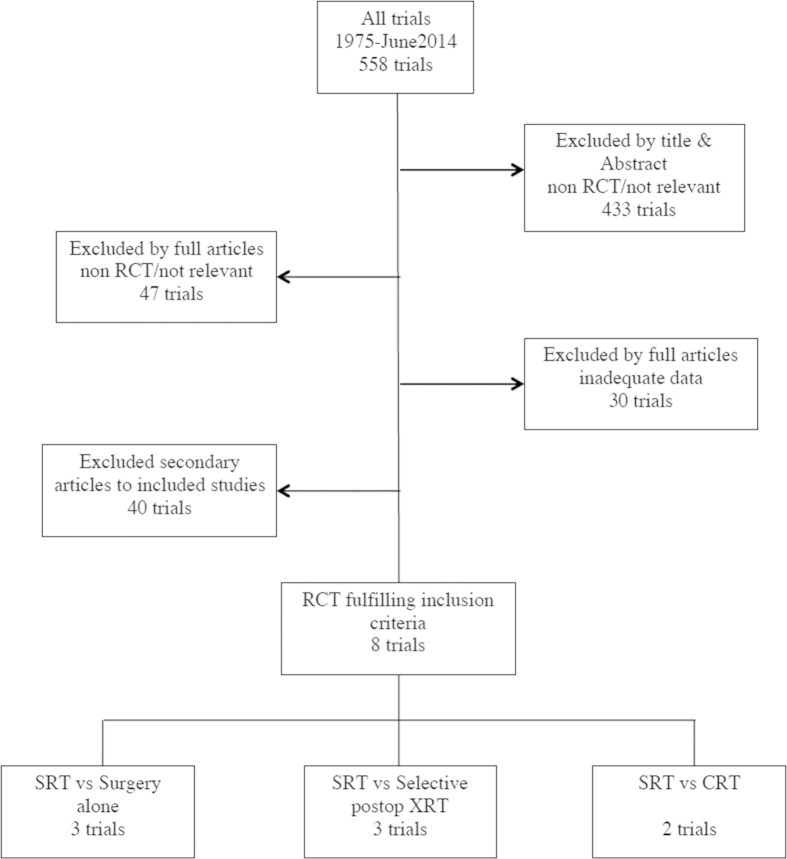
Flow chart of the literature search and study selection process.

After excluding 550 studies (selection process is shown in Fig. 1), a total of 8 trials^5^,^6^,^7^,^8^,^16^,^17^,^18^,^19^ were eligible. The regimens of included trials are listed in Table 1. Short course radiation was delivered followed by immediate surgery within 1 week in all eight trials. Three published randomized trials involving 2574 patients compared neoadjuvant short course radiation with surgery alone (Stockholm 1, Stockholm 2, Swedish trial). The Swedish RCT trial contained some patients (316) who were previously in Stockholm 2. As both studies were included in our analysis, these 316 patients have been accounted for twice. Another three published randomized trials involving 3682 patients compared neoadjuvant short course radiation with selective postoperative radiation ± chemotherapy (Uppsala, Dutch TME trial, MRC CR07). In addition, two randomized trials comparing short course radiation with long course chemoradiation group involving 638 total patients were eligible (Polish trial, TROG 01.04). The baseline characteristics of these studies and the results of assessing the quality of all included studies are listed in Table 2. Visual examination of the funnel plots with respect to all end points did not reveal any evidence of publication bias.

### SRT versus Surgery alone

**Figure 2:**
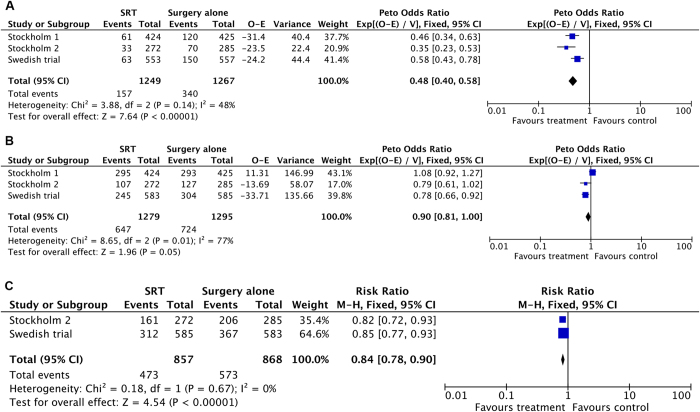
SRT versus surgery alone: (**A**) Forest plot of hazard ratio for local recurrence (**B**) Forest plot of hazard ratio for overall survival (**C**) Forest plot of risk ratio for no toxicity.

Local recurrence (Fig. 2A) was significantly improved in patients who received short course radiation compared with patients who received surgery alone. The risk of local recurrence was reduced by 52% in patients who received short course radiation (three trials, 2516 patients; HR = 0.48; 95% CI, 0.40 to 0.58; P < 0.00001). No statistically significant heterogeneity was present among these studies (I^2^ = 48%, P = 0.14).

The pooled data reached borderline significance in support for overall survival advantage in favour of SRT with an HR of 0.90 [95% CI, 0.81 to 1.00; P = 0.05] (three trials, 2574 patients; Fig. 2B). However, there was evidence of heterogeneity in the magnitude of the effect across the included studies (I^2^ = 77%; P = 0.01). Unfortunately, due to the small number of studies, a sensitivity analysis cannot be performed.

Acute toxicities after surgery were listed by type but were not graded in all three studies. The toxicities include anastomotic leak, wound dehiscence, bowel obstruction, septicaemia, wound infection, postoperative ileus and others. The proportion of patients with no toxicities post operatively favoured the surgery alone group with an RR of 0.84 [95%CI, 0.78 to 0.90; P < 0.00001] (Stockholm 2 and Swedish trial, 1725 patients; Fig. 2C). Significant heterogeneity was not evident (I^2^ = 0%; P = 0.67). Long term toxicity data were available from all 3 studies (Stockholm 1, Stockholm 2 and Swedish trial), though they each reported on different aspects of long term outcomes. Therefore, it was not possible to do a quantitative analysis across studies. A follow-up of 23 preoperatively irradiated patients and 43 non-irradiated patients from the Stockholm 1 and 2 trials revealed more frequent faecal incontinence (57%) and soiling (38%) in irradiated patients than in non-irradiated patients (26% faecal incontinence and 16% soiling)^20^. The long-term follow-up of the Stockholm trials also revealed a higher risk for small bowel obstruction (RR 1.6; 95% CI, 1.1 to 2.2), urinary incontinence, venous thromboembolism (RR 2; 95% CI, 1.2 to 3.4), and femoral neck and pelvic fractures (RR 2; 95%CI, 1.1 to 3.7) in irradiated patients when the Stockholm 1 and 2 trials were analysed together^21^,^22^. In the Swedish trial, there were more patients with increased stool frequency (20% versus 8%) and continence problems (50% versus 24%) in the SRT group^23^.

### SRT versus selective postoperative radiation ± chemotherapy

**Figure 3:**
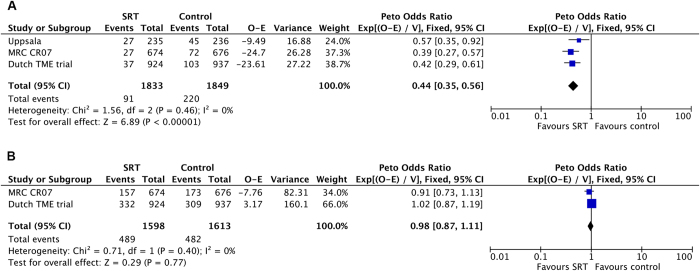
SRT versus selective postoperative radiation ± chemotherapy: (**A**) Forest plot of hazard ratio for local recurrence (**B**) Forest plot of hazard ratio for overall survival.

Local control was significantly improved, with a risk reduction of 56% for recurrence (Fig. 3A) in favour of SRT (3 trials, 3682 patients; HR = 0.44; 95% CI, 0.35 to 0.56; P < 0.00001). There was no evidence for heterogeneity between the trials (I^2^ = 0%; P = 0.46).

Data regarding overall survival were reported in two of the three trials (MRC CR07 and Dutch TME trial). Overall survival (Fig. 3B) was not significantly changed in patients who received short course radiation compared with selective postoperative radiation ± chemotherapy (two trials; HR = 0.98; 95% CI, 0.87 to 1.11; P = 0.77). There was no evidence for heterogeneity between the studies (I^2^ = 0%; P = 0.40).

Acute toxicities after surgery were only reported in the Dutch TME trial for Dutch subgroup (1530/1861 patients). The proportion of patients with no toxicities post operatively was 51.6% in the SRT group and 59.6% in the control group. Long term toxicity data were available; however, it was not possible to perform quantitative analyses across the studies. In Uppsala, there were no significant differences in bowel dysfunction, gastrointestinal disorders or urinary dysfunction between the two treatment groups^8^. During long term follow-up of the Dutch TME trial, no significant differences were observed in the rates of small bowel obstruction, sexual dysfunction, fractures and venous, arterial or cardiovascular diseases between the two patient groups^24^,^25^. However, a higher risk of bowel dysfunction was observed in the SRT group compared with the control group (62% vs 38%)^25^. Longer term follow-up (in patients with an abdomino-perineal excision) at 12 and 24 months showed that rates of small bowel obstruction, bowel dysfunction and lumbar or sacral neuropathy did not differ between the two treatment groups in the MRC CR07 trial. However, a significant increase in faecal incontinence was observed in patients treated with SRT^17^,^26^.

### SRT versus CRT

**Figure 4:**
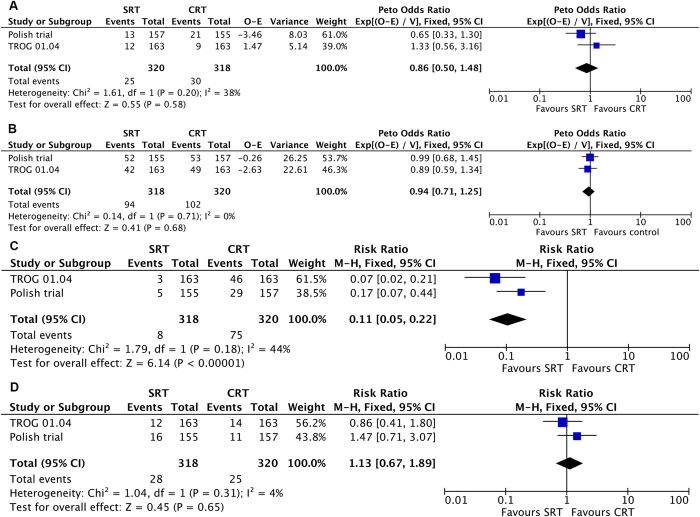
SRT versus long course radiation combined with chemotherapy: (**A**) Forest plot of hazard ratio for local recurrence (**B**) Forest plot of hazard ratio for overall survival (**C**) Forest plot of risk ratio for grade 3 or 4 acute treatment related toxicity (**D**) Forest plot of risk ratio for grade 3 or 4 late treatment related toxicity.

No statistically significant difference in local recurrence was observed in the short course irradiation group when compared with the chemoradiation group (HR 0.86; 95%CI, 0.50 to 1.48; P = 0.58; Fig. 4A), and there was no heterogeneity between studies (I^2^ = 38%, P = 0.20). In the analysis of overall survival, there was also no difference between these two groups (HR 0.94; 95% CI, 0.71 to 1.25; P = 0.68; Fig. 4B) and no heterogeneity was observed (I^2^ = 0%, P = 0.71).

In the SRT group, 8 of 318 patients (2.5%) developed a grade 3 or 4 acute treatment related toxicity, while this grade of toxicity was observed in 75 out of 320 patients (23.4%) in the CRT group. This difference was statistically significant (RR 0.11; 95% CI, 0.05 to 0.22; P < 0.00001; Fig. 4C). There was no statistically significant heterogeneity among the studies (I^2^ = 44%, P = 0.18). Grade 3 or 4 late treatment related toxicity developed in 28 of 318 patients (8.8%) treated with SRT, while this occurred in 25 of 320 patients (7.8%) treated with CRT. There was no statistically significant difference in this late toxicity rate (RR 1.13; 95% CI, 0.67 to 1.89; P = 0.65; Fig. 4D) nor was there significant heterogeneity between the studies (I^2^ = 4%, P = 0.31).

## Discussion

To our knowledge, the Colorectal Cancer Collaborative Group^4^ and Wong *et al*.^3^ conducted literature-based meta-analyses that focused on preoperative radiation, and they found beneficial effects on local control compared to surgery alone. Then, meta-analyses by De Caluwé *et al*.^27^ and Ceelen *et al*.^28^ demonstrated that preoperative CRT improves local control in resectable stage II and III rectal cancer compared to preoperative RT alone. None of the previous meta-analyses analysed short course radiation separately. Although short course radiation for rectal cancer has been investigated over the past two decades, many clinicians in the United States and China have not adopted a short course radiation approach. Nevertheless, we comprehensively searched for literature examining the outcomes after short course radiation.

In this systematic review of data from 6894 patients in 8 trials of short course radiation for rectal cancer, short course radiotherapy substantially reduced the risk of local recurrence compared with surgery alone or selective postoperative radiation ± chemotherapy. In the past, some clinicians questioned the results in Stockholm trials and Swedish trial as the surgery used in these slightly older studies was not consistent with optimal current surgical plans. However, the effect on reducing local recurrence remains consistent after implementation of the total mesorectal excision (TME) technique in the Dutch TME trial and MRC CR07 trial. The effect is also comparable when compared with long course chemoradiation, which is in contrast with the meta-analysis findings by De Caluwé *et al*.^27^and Ceelen *et al*.^28^ that demonstrated a significant benefit in local control by adding chemotherapy to radiotherapy. The aforementioned meta-analyses also included studies that compared conventionally fractionated preoperative radiation with long course chemoradiation in patients with resectable rectal cancer. As uncontrolled local recurrence can have a destructive effect on a patient’s quality of life, the improved local control with SRT might be a sufficient benefit to justify its use.

Although SRT has been shown to marginally improve survival over surgery alone in our meta-analysis, it must be emphasized that the Swedish trial was the only included trial that reported a significant improvement in survival, and there was evidence of heterogeneity across the included studies. Therefore, this result should be interpreted with caution. Contrary to the belief that reduction in local recurrence after radiation increases the likelihood of overall survival, no significant effect in terms of OS was found from SRT approach compared with selective postoperative radiation ± chemotherapy. The result was similar with prior meta-analyses^3^,^4^,^27^,^28^. This finding seems to be reasonable, considering the following facts. First, the reduction in mortality from rectal cancer may be largely counterbalanced by the increased risk of death from other causes. Second, the number of patients may not be large enough to reliably detect small but significant differences in long-term survival. Furthermore, we did not find a statistically significant difference in overall survival between the SRT group and the long course chemoradiation group.

It was reported in several studies that SRT is associated with an increased risk of certain toxicities at long-term follow-up compared with patients treated with surgery alone or selective postoperative radiation. Due to significant improvements in radiation treatment plans and because of different radiation techniques and irradiated volumes, it is difficult to interpret the results of toxicity across different studies. However, treatment related toxicity was generally acceptable as evidenced by the relatively high compliance rates in the mentioned studies. When compared with CRT, grade 3 and 4 acute treatment related toxicity was more pronounced in the CRT group. With respect to late toxicity, no significant differences were observed between patients who received short course radiotherapy and those who received chemoradiotherapy. However, the duration of follow-up in both trials was too short to draw definitive conclusions regarding late adverse effects. Longer follow-up is required to fully assess late effects.

Finally, what are the implications of these results for current practice? On one hand, SRT has certain advantages compared to long course radiation, including a small number of fractions that makes short course radiation less expensive, more convenient and improves compliance. On the other hand, because cancer cells sterilized by radiation require time to undergo necrosis^29^, short course radiation with immediate surgery has limited effect on tumour shrinkage and downstaging. However, the problem may be solved by short course radiation with delayed surgery. An interim analysis of the Stockholm III trial comparing 3 preoperative radiotherapy schemes showed a pathologic complete response (pCR) in 12.5% of patients in the short course radiation with delayed-surgery group whereas the rate of pCR after short course radiotherapy with immediate surgery was only 0.8%. As shown in other nonrandomized studies^30^,^31^, the rate of pCR after short course radiation and delayed surgery is not largely different from that which is observed after conventionally fractionated chemoradiation. Therefore, short course radiation with a delayed interval to surgery can be a reasonable option for elderly patients with comorbidities, who are often unfit for chemotherapy and can be used when tumour shrinkage is necessary prior to resection.

In addition to a limited effect on tumour shrinkage and downstaging, short course radiation was called into question given its inability to safely be combined with adequate doses of systemic chemotherapy. As has been shown previously, the local efficacy of 5 × 5Gy and conventionally fractionated chemoradiation is similar. However, the dose of chemotherapy must be reduced in order to keep the level of toxicity acceptable. This may negatively affect the systemic efficacy of chemotherapy. Therefore, short course radiation with sequential consolidation chemotherapy during the interval to surgery may enable intensification of both systemic treatment and radiation. It is feasible and promising particularly in patients with a rectal cancer and potentially resectable synchronous metastatic disease. In a study reported by van Dijk *et al*.^32^, 48 patients with rectal cancer and synchronous distant metastases received such management. The result was promising with a complete pathologic response in 22.5% of patients and curative surgery in 85% of patients. Prospective studies to validate this treatment strategy are needed in the future.

This analysis had specific limitations. First, our meta-analysis is not based on individual patient data and therefore differences in individual baseline characteristics cannot be integrated. The second limitation is the relatively small number of enrolled patients. Finally, but importantly, the duration of follow-up for some studies was too short to draw definitive conclusions. Regardless of the limitations, this meta-analysis demonstrates that SRT provided significant benefit for resectable rectal cancer patients in local control, the effect of which is equivalent with long course chemoradiation. However, SRT does not benefit overall survival when compared with either selective postoperative radiation ± chemotherapy or long course chemoradiation. SRT related toxicity was generally acceptable and SRT decreased short-term toxicity compared to preoperative chemoradiation in resectable rectal cancer. In conclusion, SRT is a reasonable alternative for resectable rectal cancer patients and should be part of an informed discussion of treatment options for this group.

## Additional Information

**How to cite this article**: Chen, C. *et al*. Short Course Radiation in the Treatment of Localized Rectal cancer: A Systematic Review and Meta-Analysis. *Sci. Rep*. **5**, 10953; doi: 10.1038/srep10953 (2015).

## Figures and Tables

**Table 1:** Trials studying short course radiation in localized rectal cancer.

Study	Regimen	No. of Patients
Stockholm 1	Arm 1: 5×5 Gy with immediate surgery	424
	Arm 2: surgery alone	425
Stockholm 2	Arm 1: 5 ×5 Gy with immediate surgery	272
	Arm 2: surgery alone	285
Swedish trial	Arm 1: 5×5 Gy with immediate surgery	585
	Arm 2: surgery alone	583
Uppsala	Arm 1: 5 ×5.1 Gy with immediate surgery	235
	Arm 2: selective postoperative radiotherapy for patients with stage B/C, 60 Gy in 30 fr	236
Dutch TME trial	Arm 1: 5 ×5 Gy with immediate surgery	924
	Arm 2: selective postoperative radiotherapy for patients with positive margins (11%), 50.4 Gy in 28 fr	937
MRC CR07	Arm 1: 5 ×5 Gy with immediate surgery	674
	Arm 2: selective postoperative CRT for patients with positive circumferential margin(9%), 45 Gy in 25 fr +5-FU	676
Polish trial	Arm 1: 5 ×5 Gy with immediate surgery	155
	Arm 2: 50.4 Gy in 28 fr with concomitant CT weeks 1 & 5	157
TROG 01.04	Arm 1: 5 ×5 Gy with immediate surgery	163
	Arm 2: 50.4 Gy and 5-FU 225 mg/m^2^/day followed by surgery at 4-6 weeks	163

**Table 2:** Baseline characteristics of patients included in the meta-analysis.

Study	Year	Stage	Criteria used to define rectal cancer	Median follow-up(year)	Surgery	Quality score
Stockholm 1	1980–1987	Any stage resectable	below sacral promontory	4.5	AP/anterior resection	0.74
Stockholm 2	1987–1993	Any stage resectable	below sacral promontory	9	AP/anterior resection	0.88
Swedish trial	1987–1990	T1-3	below sacral promontory	13	AP/anterior resection	0.82
Uppsala	1980–1985	Dukes A/B/C	NA	Minimum 5	AP/anterior resection	054
Dutch TME trial	1996–1999	Any stage resectable	15 cm from anal verge, below S1-2	12	AP/anterior resection with TME technique	0.88
MRC CR07	1998–2005	I-III	NA	4	APR/non-APR with TME technique	0.83
Polish trial	1999–2002	T3 or resectable T4	inferior edge palpable of digital exam	4	AP/anterior resection or Hartman with TME	1
TROG 01.04	2001–2006	cT3N any	within 12 cm of the anal verge	5.9	APR/non-APR with TME technique	0.81
